# A cyanosulfidic origin of the Krebs cycle

**DOI:** 10.1126/sciadv.abh3981

**Published:** 2021-08-13

**Authors:** Dougal J. Ritson

**Affiliations:** MRC Laboratory of Molecular Biology, Francis Crick Avenue, Cambridge Biomedical Campus, Cambridge CB2 0QH, UK.

## Abstract

The centrality of the Krebs cycle in metabolism has long been interpreted as evidence of its antiquity, and consequently, questions regarding its provenance, and whether it initially functioned as a cycle or not, have received much attention. The present report shows that prebiotic oxidation of α-hydroxy carboxylates can be achieved by UV photolysis of a simple geochemical species (HS^−^), which leads to α-oxo carboxylates that feature in the Krebs cycle and glyoxylate shunt. Further reaction of these products leads to almost all intermediates of the Krebs cycle proper, succinate semialdehyde bypass, and glyoxylate shunt. Fumarate, the missing Krebs cycle component, and the required α-hydroxy carboxylates can be provided by a highly related hydrogen cyanide chemistry, which also provides precursors for amino acids, nucleotides, and phospholipids.

## Teaser

All four cellular subsystems can be provided under compatible prebiotic conditions.

## INTRODUCTION

The metabolic subsystem of cells links, provisions, and powers the other three cellular subsystems, which in turn, encode, catalyze, and encapsulate it, and the way in which this complex synergistic ensemble arose at the dawn of life is a major enduring enigma. A few years ago, a potentially prebiotic and common origin of nucleotides, amino acids, and lipid precursors was reported, which relied upon the photochemical reduction of hydrogen cyanide (and products derived therefrom) using hydrosulfide (HS^−^) as the stoichiometric reductant (*1*, *2*), thereby challenging traditionally held views that one cellular subsystem must have arisen before all others (*3*–*5*). Core components of metabolism were largely absent, however, and would presumably be required at the onset of biology to replenish the depleted stocks of life’s foundational molecules, e.g., amino acids consumed during oligopeptide (bio)synthesis, consequentially developing (proto)biosynthetic pathways in the process. Accordingly, the cyanosulfidic network was reevaluated to see whether missing constituents or links to the metabolic subsystem could be found with the added constraint that the chemistry should be compatible with that used to form the other three cellular subsystems (*2*).

**Fig. 1:**
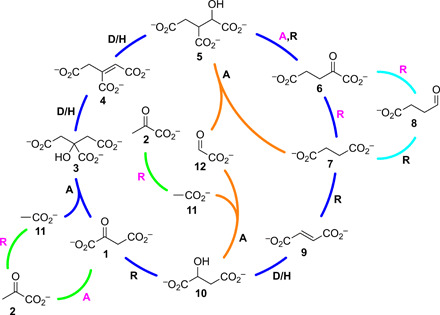
Topology of the Krebs cycle (dark blue) as precursed (green) by pyruvate 2 with glyoxylate shunt (orange) and succinate semialdehyde bypass (cyan). For the sake of simplicity, thioester derivatives of certain core metabolites are not shown. In the Krebs cycle, citrate **3** is converted to aconitate **4** and thence isocitrate **5** by dehydration-hydration, and the latter compound then undergoes oxidation and decarboxylation to α-ketoglutarate **6**. Decarboxylation and oxidation of α-ketoglutarate **6** to succinate **7** can occur either directly or indirectly, via succinate semialdehyde **8**, in a variant of the cycle (*37*). Dehydrogenation of succinate **7** then generates fumarate **9,** which is hydrated to malate **10**. The cycle is closed by aldol/Claisen-type reaction of acetate **11** with oxaloacetate **1**, the oxidation product of malate **10**. In the glyoxylate shunt (*38*), isocitrate **5** is cleaved by retro-aldol/Claisen-type reaction into succinate **7** and glyoxylate **12**, and the latter is converted to malate **10** by aldol/Claisen-type reaction with acetate **11**. In both the Krebs cycle proper and the glyoxylate shunt, the acetate **11** input is provided by oxidative decarboxylation of pyruvate **2**. The cycle operates in the reductive sense by essentially reversing the steps; however, the epicycle (bottom left) renders the system autocatalytic due to the fact that **11** (cleaved from citrate as acetyl–coenzyme A) can undergo reductive carboxylation furnishing pyruvate **2**, which can then reenter the Krebs cycle after carboxylation. R, redox reaction; D/H, dehydration/hydration; A, aldol/Claisen-type reaction; magenta letters denote that CO_2_ is involved in this step.

In the reported cyanosulfidic network (*2*), 12 amino acids are accessed via Strecker synthesis, a reaction that proceeds through an amino acid’s corresponding amino nitrile (*6*). Until the first step of hydrolysis takes place, Strecker synthesis is under the control of equilibria, and at any time or location on early Earth when the concentration of ammonia or the pH was relatively low, amino nitriles would be partially, or wholly, converted to cyanohydrins [with the exception of proline amino nitrile; (*2*)]. The final hydrolysis products of cyanohydrins are the corresponding α-hydroxy carboxylates, and consequently, a means of oxidizing α-hydroxy carboxylates to α-oxo carboxylates would provide the penultimate compounds featured in numerous amino acid biosyntheses and suggest a link to extant metabolic pathways. In addition, four of those α-oxo carboxylates (oxaloacetate **1**, pyruvate **2**, α-ketoglutarate **6**, and glyoxylate **12**; fig. S1) are constituents of the Krebs cycle or its variants (Fig. 1), viewed by some as the basis from which all metabolism was built [as representative examples, see (*7*–*10*)]. The most recent endeavors to unravel the foundation of the Krebs cycle have focused on reaction networks created from aldol chemistry of **2** and **12**, and an oxidative synthesis of these starting materials under a unified set of conditions would alleviate problems regarding previously suggested and disparate sources of pyruvate **2** and glyoxylate **12** (*10*, *11*). In particular, the reduction of CO_2_ to **2** or **12** occurs under different (*12*–*15*), and in some cases geochemically unlikely [(*15*); see (*16*, *17*)], conditions, and the survival of each of these compounds under conditions of the other’s synthesis has not been demonstrated. Thus, a prebiotic means of oxidizing α-hydroxy carboxylates to α-oxo carboxylates was sought.

## RESULTS

### Oxidation of α-hydroxy carboxylates

**Fig. 2:**
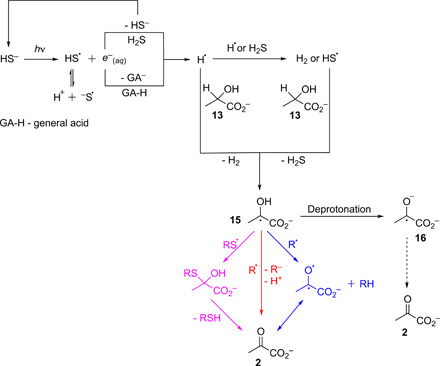
Possible mechanisms for oxidation of lactate 13 (and other α-hydroxy carboxylates) by HS^−^/UV light. Note that reaction arrows are depicted as unidirectional for simplicity. R = H(S)_n_, for example. Previously, it was described in detail [see (*18*) , including Supplementary Discussion 2 of (*18*)] that H^•^ production via irradiation of solutions of HS^−^ at pH 6.5 is expected to be rapid alongside the production of HS^•^, and a simplified view is depicted in the top left. Presumably, the first step of the oxidation of **13** involves hydrogen atom abstraction from the α-position of **13**, which could be performed by H^•^ or HS^•^, resulting in the captodatively stabilized radical **15**. Lactate radical **15** can then undergo recombination with a sulfur-centered radical (magenta pathway), with ensuing loss of the corresponding thiol/sulfide to give pyruvate **2**, or hydrogen atom abstraction from the alcohol group of **15** could produce the ketone of **2** (blue pathway). Electron transfer to R^•^ could also occur, e.g., disproportionation or reduction of a thiyl radical, which would give **2** after deprotonation of the alcohol (red pathway). Although oxidation of the ketyl radical anion **16**, formed from **15**, cannot be ruled out, it would seem less likely given that the p*K*_a_ (where *K*_a_ is the acid dissociation constant) of the alcohol of **15** is ~9.8 (*39*) and the reactions were run at pH 6.5. Captodative stabilization of the transition state seems to be necessary for H^•^ or HS^•^ to be able to access the reaction manifold, as attempted oxidation of isopropanol under the same conditions did not yield acetone. It has been reported that H^•^ is capable of abstracting the α-proton from α-hydroxy acids, albeit at low pH where the acids were present in protonated form (*39*).

Recently, it was found that reduced phosphorus species could be oxidized to the level of phosphate by two environmental inputs which are also used for photochemical reduction of hydrogen cyanide—HS^−^ and ultraviolet (UV) light (*18*). Therefore, the oxidation of lactate **13**, glycolate **14**, malate **10**, and α-hydroxyglutarate **18** to the corresponding carbonyl compounds using the same, or similar, conditions was investigated (fig. S1). In the first instance, lactate **13** (60 mM) was irradiated in the presence of NaSH (60 mM) and phosphate buffer (100 mM) at pH 6.5 with low-pressure Hg lamps (principal emission, 254 nm), and the progress of the reaction was monitored by ^1^H nuclear magnetic resonance (NMR) spectroscopy. Reaction was reasonably rapid, with pyruvate **2** being observed (~4% yield) after 1 hour, and after 2.5 hours, **2** was present in ~15% yield (fig. S2). Increasing the concentration of NaSH twofold actually slowed the reaction, with ~1% of **2** being present after 1-hour reaction and ~3% yield after 2.5 hours (fig. S2). Assuming that hydrogen atoms (H^•^) and/or the hydrosulfide radical (HS^•^, or its anion, ^−^S^•^) were responsible for induction of the reaction, it seemed likely that higher concentrations of hydrosulfide, and therefore H^•^ and HS^•^, were detrimental, as recombination of H^•^ radicals would lead to loss of H_2_ and recombination of HS^•^ radicals would lead to formation of polysulfides and eventually S_8_. However, as time progressed and S_8_ precipitated, the concentration of HS^−^ in solution would decrease and this would allow more productive reaction of H^•^ and/or HS^•^ with the substrate later in the reaction. Thus, the observed increase in rate of oxidation in the first reaction, i.e., ~4% yield for the first 60 min, compared to 11% yield for the next 90 min (fig. S2, spectra A and B), can be accounted for. Accordingly, the amount of NaSH was reduced to a substoichiometric level (25 mM), which then provided ~12% of **2** after 1 hour and ~26% of **2** after 2.5 hours (fig. S3). Whether the solvent had been purged with argon or not had little effect on the outcome of the reaction (fig. S4). The rapid onset of oxidation and linear increase in yield of **2** with time (fig. S5) suggest that polysulfide formation, thought to be required for the oxidation of phosphite under similar conditions (*18*), was not responsible for the oxidation of 2, and mechanistic rationales such as those depicted in Fig. 2 are more likely.

**Fig. 3:**
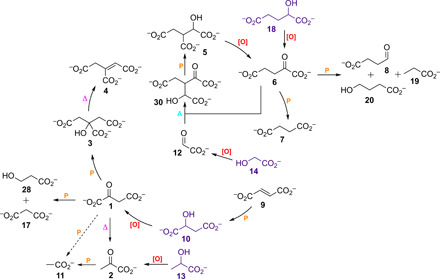
Summary of the key transformations observed during the present study. The purple molecules represent the key inputs from which all other intermediates can be accessed, except fumarate **9**. It was pointed out that **9** is converted to **10** by photolysis in aqueous solution (*20*), which was confirmed in this study and was found to proceed via 3-carboxy β-lactone (figs. S23 and S24): A, aldol in phosphate or carbonate buffer; Δ, thermal reaction; [O], NaSH/*h*ν; P, *h*ν.

Higher yields of **2** could not be obtained, as **2** was able to undergo its own photochemistry when concentrations became high enough to allow competition with NaSH (and/or polysulfides) for absorption of UV light (fig. S6). Although phosphate improved the yield of the reaction, it was not necessary for successful oxidation, with ~13% of **2** being obtained in the absence of phosphate after 2.5 hours under analogous conditions (fig. S7). Reducing the concentration of hydrosulfide further (5 mM), which may be more geologically relevant (*19*), the oxidation of **13** still proceeded well, giving **2** in ~17% yield after 1 hour alongside the production of what appeared to be 2,3-dimethyl tartrate ~15% (fig. S8). When the concentration of lactate **13** was also lowered (5 mM), the rate and yield of the reaction actually increased (~29% yield of pyruvate **2** after 1 hour; fig. S8). The oxidation of glycolate **14** gave glyoxylate **12** in a lower yield of ~4% (Fig. 3 and figs. S9 and S10). This may reflect the reduction in stabilization, compared to **13**, of the less substituted hydroxyalkyl radical (/ketyl radical anion), which is formed after initial hydrogen atom abstraction (and proton loss) from **14**. Alternatively, it may represent a shift in the position of redox equilibrium between carbonyl compound and alcohol. Some evidence for this was obtained by comparing the reduction of glyoxylate **12** and pyruvate **2** by HS^−^ under photochemical conditions. The reduction of **12** gave ~12% of glycolate **14** after 1-hour reaction, whereas only ~1% reduction of **2** to lactate **13** was observed under the same conditions (figs. S11 and S12). When malate **10** was subjected to the oxidation conditions, oxaloacetate **1** was obtained (~4% yield; Fig. 3 and fig. S13), but higher yields of this compound could not be achieved as β-decarboxylation of **1** to **2**, followed by Norrish type-I cleavage and oxidation to acetate **11**, was competitive with oxidation of **10**, and, although slower, α-cleavage and oxidation of **1** gave malonate **17** (Fig. 3). Oxidation of α-hydroxyglutarate **18** under the standard conditions produced α-ketoglutarate **6** in ~20% yield after 2.5-hour irradiation, with simultaneous Norrish type-I chemistry producing succinate **7** in ~2% yield (Fig. 3 and fig. S14). When the photochemistry of **6** was examined more closely, it was found that after 4-hour irradiation, **7** was present in ~23% yield, succinate semialdehyde **8** in ~12% yield, and propionate **19** in ~4% yield (Fig. 3 and figs. S15 and S16). Given the formation of **19** from **6**, it would seem likely that acetate **11** can also be derived photochemically from oxaloacetate **1** itself, in addition to the indirect formation of **11** after β-decarboxylation of **1** to pyruvate **2** and subsequent photochemical conversion of **2** to **11** (Fig. 3). Irradiation of succinate **7** under the same conditions did not afford **8**, confirming that **7** stems from the photochemistry of **6** (fig. S17). To determine whether **8** could be made in one pot, α-hydroxyglutarate **18** was irradiated in the presence of NaSH for a prolonged period of time, and after 10-hour reaction, **7** was present in ~10% yield, **8** in ~23% yield, propionate **19** in ~4% yield, and 4-hydroxybutyrate **20** in ~4% yield (Fig. 3 and figs. S18 and S19). The idea that **8** derived from **6** and not from the reduction of succinate **7** by photolysis of HS^−^ was then confirmed (fig. S20). Given that there is one other oxidizable α-hydroxy carboxylate in the Krebs cycle itself, isocitrate **5**, this compound was also subjected to the oxidation conditions. On shorter time scales of irradiation, α-ketoglutarate **6** and succinate **7** were observed, whereas **7** and succinate semialdehyde **8** predominated on longer time scales of irradiation (Fig. 3 and figs. S21 and S22). The oxidation of **5** presumably gives oxalosuccinate, which then undergoes spontaneous decarboxylation to afford **6**.

### Elaboration of α-oxo carboxylates into further Krebs cycle intermediates

**Fig. 4:**
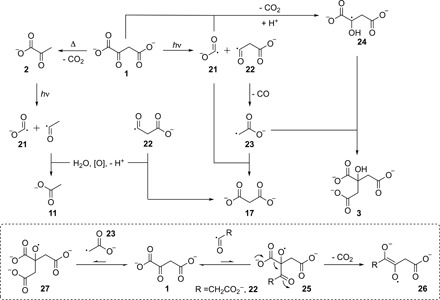
Mechanistic considerations of the photochemical synthesis of citrate 3 from oxaloacetate 1.

Although citrate **3** was obtained in the study of Stubbs *et al.* (*11*), the high concentration of oxaloacetate **1** required would seem out of reach of the present chemistry given the modest yield of **1** and its thermal instability, which disallows concentration. The photochemical conversion of oxaloacetic acid into citric acid at low pH has been described by Waddell *et al.* (*20*), although a prebiotic synthesis of oxaloacetic acid was lacking from those studies. As this reaction is also driven by UV light, it was hoped that it would be possible to oxidize malate **10** to oxaloacetate **1** and then have **1** react photochemically in situ to produce **3**, thus avoiding the thermal instability issues related to **1**. In the first instance, Waddell’s reaction was repeated but under conditions closer to those used here [oxaloacetate **1** (5 mM), phosphate buffer (50 mM), pH 6.5, low-pressure Hg lamps], and after 1 hour, **1** was almost fully consumed and citrate **3** had been formed in ~20% yield alongside malonate **17** (~18% yield), pyruvate **2** (~2% yield), and acetate **11** (~14% yield; Fig. 3 and fig. S25), which may be explained by the mechanism outlined in Fig. 4. It is assumed that the reaction proceeds with homolytic cleavage of the C1-C2 bond in oxaloacetate **1** producing the carboxyl radical anion CO_2_^•−^ **21** and acyl radical **22**, which is in accord with previous electron paramagnetic resonance (EPR) studies on the radical intermediates of α-oxocarboxylate photolysis (*21*, *22*). Acyl radical **22** can undergo decarbonylation producing the relatively stable carboxymethyl radical **23**, which may then recombine with the carboxyl radical anion **21** forming malonate **17**. Alternatively, **17** can be produced by hydration and oxidation of acyl radical **22**. The carboxyl radical anion **21** can reduce oxaloacetate **1**, giving, after ketyl radical anion protonation, the malate radical **24**. Radical recombination of **24** with the carboxymethyl radical **23** then produces citrate **3**. In studies of the photolysis of oxaloacetate **1**, addition of acyl radical **22** to the α-carbonyl of another molecule of starting material **1**, giving a transient alkoxy radical **25**, was inferred (dashed box) (*21*). Rapid β-decarboxylation of this alkoxy radical was suggested as the means by which semidione radical anion **26**, which was characterized by EPR spectroscopy, was formed (*21*). Subsequent reduction or oxidation of **26** would give an α-hydroxy carbonyl compound or a 1,2-dicarbonyl compound, neither of which are stable under UV photolysis conditions (*23*), and this may be why they were not obviously apparent in the ^1^H NMR spectra obtained. The addition of acyl radical **22** to oxaloacetate **1** suggests that the carboxymethyl radical **23** might also add to **1**, in which case alkoxy radical **27** would be produced. Waddell *et al.* (*20*) suggested that citrate might arise in this way by subsequent reaction of **27** with a hydrogen atom. However, the addition of radicals to carbonyl groups is reversible and unfavorable unless followed by a rapid exothermic step such as decarboxylation to a delocalized radical. Alkoxy radical **27** has no such intramolecular path available to it and so is expected to revert to oxaloacetate **1** and carboxymethyl radical **23** faster than it would undergo an intermolecular reaction.

The one-pot conversion of malate **10** to citrate **3** under standard conditions was then attempted, but after 4- or 7.5-hour irradiation, **3** could not be observed by routine ^1^H NMR spectroscopy, although oxaloacetate **1** was present, as were malonate **17** and acetate **11**, demonstrating that the requisite Norrish type-I chemistry was taking place (fig. S26). Reasoning that hydrosulfide and/or polysulfide by-products were somehow interfering with the desired reaction, the previous irradiation of **1** in phosphate buffer was repeated in the presence of NaSH (25 mM), after which **3** could not be observed by routine ^1^H NMR spectroscopy (fig. S27). As prolonged UV irradiation of HS^−^ forms polysulfide species and ultimately S_8_, which is insoluble in H_2_O, the oxidation of malate **10** was carried out for 24 hours and yielded citrate **3** ~4%, 3-hydroxypropionate **28** ~9%, malonate **17** ~5%, acetate **11** ~25%, and what was presumably EtOH ~8% (Fig. 3 and figs. S28 and S29). However, given the relatively rapid photooxidation of NaSH (effective oxidation of substrates was typically over in 3 to 4 hours) coupled with the fact that, after 7.5 hours of irradiation, **3** could not be seen in the ^1^H NMR spectrum and the concentration of **1** was being depleted (fig. S26), it was not clear that the formation of **3** was due to the last traces of **1** undergoing the Waddell chemistry or something else. It seemed feasible that precipitated S_8_ was being photolyzed later in the reaction to liberate low levels of thiyl radicals, which could then oxidize **10** to **1**, yet were in concentrations low enough not to interfere with the Waddell chemistry. Consequently, malate **10** was irradiated in phosphate buffer in the presence of elemental sulfur for 24 hours, after which time citrate **3** was present in ~3% yield (fig. S30). Under the same conditions without added S_8_ or NaSH, **3** was not unambiguously detected, but if present, it was formed in a maximum yield of ≤0.4% (figs. S31 and S32). The other α-hydroxy acids showed similar reactivity, giving low or trace amounts of products when compared to reactions with NaSH included (figs. S33 to S35). If the α-oxo acid salts could be observed when NaSH was absent from the reaction, they were present in <0.5% yield.

The thermal conversion of citric acid via aconitic acid to isocitric acid has also been reported (*24*), but repetition of this reaction returned *cis*-aconitate **4** as the major product (~23% yield) with a lesser amount of *trans-*aconitate **29** (~3% yield) and isocitrate **5** could not be detected by routine ^1^H NMR spectroscopy (Fig. 3 and fig. S36). Whether the sample was heated in the dry state or dissolved in H_2_O first essentially had no effect on the reaction (figs. S36 and S37). Therefore, photochemical routes to **5** were considered, and in the same way that irradiation of α-ketoglutarate **6** had furnished succinate **7**, an α-keto homolog of **5** could be expected to undergo oxidative α-decarboxylation to yield **5**. It quickly became apparent that one of the three possible congeners (**30**) was an aldol product of α-ketoglutarate **6** and glyoxylate **12** (Fig. 3). Therefore, **6** (50 mM) was incubated with **12** (55 mM) in either phosphate (pH 6.5) or carbonate (pH 10.0) buffer (100 mM) at 60°C for the desired amount of time. The reaction in carbonate buffer was rapid, giving ~69% of **30** after 1 hour and ~3% of the dehydrated aldol product **31** (fig. S38), whereas the reaction at neutral pH was slower and gave ~10% of the aldol product **30** and ~3% of **31** after 24 hours (fig. S38). After irradiation of the crude reaction containing **30** for 11 hours, isocitrate **5** had formed in ~10% yield (fig. S39). Although fumarate **9** was not obtained during the chemistry investigated here, it is a product of the cyanosulfidic chemistry that also generates the starting α-hydroxy carboxylates (fig. S1) (*2*).

### Simultaneous oxidative and reductive synthesis

The fact that HS^−^ and UV light are key requirements for the oxidation of α-hydroxy carboxylates and the reductive chemistry of HCN begged an obvious question: If HCN was still supplied to the environment in which nitrile hydrolysis had taken place, does reductive or oxidative chemistry occur? Therefore, the reaction of lactate **13** with NaSH was repeated but, this time, in the presence of HCN. A very clean reaction was observed, and pyruvate cyanohydrin **32** was formed in ~54% yield, with pyruvate **12** and pyruvate hydrate **12-h** present in ~9% combined yield, but it was also found that HCN had undergone reductive homologation, giving rise to glycolonitrile **33** and glycolaldehyde cyanohydrin **34** (fig. S40). Oxidation of malate **10** under the same conditions gave oxaloacetate cyanohydrin **35** in ~23% yield, and again, **33** and **34** were formed (fig. S41). The oxidation of α-hydroxyglutarate **18** and glycolate **14** was also significantly enhanced by inclusion of cyanide with **18** leading to α-ketoglutarate **6** (~6% yield) and α-ketoglutarate cyanohydrin **36** (~45% yield) and **14** giving glyoxylate cyanohydrin **37** (~20% yield), again with the concomitant formation of **33** and **34** (figs. S42 to S44). When the system was challenged further by using an excess of HCN, the yields were little perturbed (figs. S45 and S46). The improved yields of oxidized products relative to the analogous reactions in the absence of HCN are presumed to be due to the effective removal of the chromophore of the α-oxo carboxylate by addition of HCN to the α-carbonyl, thereby suppressing photolysis of the α-oxo carboxylate (see fig. S6) and, in the case of oxaloacetate **1**, also protecting against β-decarboxylation. Somewhat surprisingly, citrate **3** was still formed in the presence of HCN and HS^−^ under UV irradiation (~2% yield after 22-hour irradiation; fig. S47). At times on early Earth when the environment became depleted in cyanide, the carbonyl compounds would have been reformed from their cyanohydrins by the slow loss of HCN (*25*), with the exception of oxaloacetate cyanohydrin **35**, which would be expected to return pyruvate **2** (figs. S48 to S50). In an attempt to replicate more realistic conditions, the oxidation of lactate **13** (60 mM) was performed in the presence of HCN (45 mM) with the cuvette left open to the atmosphere. After 2.5-hour reaction, pyruvate cyanohydrin **32** was still formed in ~30% yield alongside ~14% of **2**, which at least suggests that the reaction may have been feasible in a geologic setting (fig. S51). It should be noted that the cooling fans of the UV apparatus as well as prolonged exposure of the reaction to atmospheric O_2_ under photochemical conditions are liable to have affected the outcome of this reaction (*26*). The first products of HCN reductive homologation, glycolonitrile **33** and glycolaldehyde cyanohydrin **34**, were formed in all experiments that included HCN, in concentrations of ~2 to 5 mM and ~1 to 2 mM, respectively (figs. S40 to S47 and S51). Thus, HS^−^ photoredox chemistry can simultaneously synthesize α-oxo acid salts by oxidation while reducing HCN to gateway molecules for the cyanosulfidic synthesis of amino acid, lipid, and RNA precursors (*2*). Noteworthy is the fact that amino acids appear to be almost inert under the oxidative conditions used, which may have allowed the selective oxidation of α-hydroxy acids in the presence of amino acids on primitive Earth (figs. S52 and S53). The unreactive nature of alanine under these conditions is somewhat surprising considering that the photolysis of amino acids has been reported previously (*27*, *28*). Thus, alanine was subjected to irradiation at high (~9) and low (~3) pH in the absence of NaSH. At low pH, alanine was stable, with only ~3% photolysis products after 2.5-hour irradiation (fig. S54). Under the high pH reaction conditions, alanine was somewhat more susceptible to photolysis, giving a total of ~6% of products after 2.5-hour reaction (fig. S54). Inclusion of NaSH in the low pH reaction effectively prevented any reaction of alanine, even after 2.5-hour irradiation (fig. S55). At high pH, the photochemical decomposition pathways of alanine were again inhibited, but now, the slow production of pyruvate **2** could be observed (fig. S55). This appeared to stem from oxidation of alanine, but the oxidation of a trace amount of lactate **13**, formed in situ, cannot be ruled out (*27*). Last, the photochemical oxidation of **13** at high and low pH was examined. At pH ~3, small amounts of **2** were obtained, but most significant was the high conversion of **13** to EtOH after 2.5 hours (~12%; fig. S56). It should be noted, however, that a substantial conversion of **13** to EtOH at low pH was also found to take place in the absence of added NaSH (~7% after 2.5 hours; fig. S57). At high pH (~9), the oxidation of **13** in the presence of NaSH was much slower but gave little else besides **2**, although at a much-reduced rate compared to the same reaction run at circumneutral pH (~3% of **2** after 2.5 hours; fig. S56).

## DISCUSSION

Using an oxidative approach that requires HS^−^ and UV light, four α-hydroxy carboxylates have provided access to all the intermediates of the Krebs cycle, its succinate semialdehyde bypass, and the glyoxylate shunt, with the exception of fumarate **9**. The four α-hydroxy carboxylates and **9** can be provided by a cyanosulfidic network that involves the reduction of hydrogen cyanide and nitriles by HS^−^ and UV irradiation (*2*). In addition, two of the oxidation products are pyruvate **2** and glyoxylate **12**, used as starting materials in proposed abiotic analogues of the Krebs cycle (*10*, *11*). The incompatibility of ferrous ions (*10*) with HS^−^ (*29*) would seem problematic, however, or demand separation of these two chemistries, but the putative metal-free ancestral Krebs cycle (*11*) could potentially have overlapped with the current oxidative chemistry. Although H_2_O_2_ (*11*) is also incompatible with HS^−^ (*30*), its requirement is obviated by the chemistry now described. Possibly, the biggest unknown for the reported chemistry is the UV light source; the low-pressure Hg lamps used in this study emit the bulk of their output at 252 to 256 nm, which does not represent the spectral radiance expected to reach Earth’s surface from the young Sun, nor its UV flux at those wavelengths (*31*). Although it has been considered a reasonable proxy for the young Sun at these wavelengths (*31*), a much more detailed investigation would be useful to determine planetary time scales of the reactions (*32*).

Although the (reverse) Krebs cycle is not expected to have been operational before the advent of (ribo)enzymic catalysis (*33*, *34*), the presence of pyruvate **2**, α-ketoglutarate **6**, and glyoxylate **12** would offer a facile advantage for oligopeptide production if promiscuous transaminase activity was found at the onset of biology. Once the environment was depleted in α-oxo carboxylates, a new selective pressure would develop, and an oxidoreductase activity would circumvent this stranglehold by providing **2**, **6**, and **12** from lactate **13**, α-hydroxyglutarate **6**, and isocitrate **5** and glycolate **14**, respectively. If any individual’s transaminase activity had evolved to operate at a sufficient rate to compete with decarboxylation of oxaloacetate **1**, or at the time when it did, aspartate also would be freely available to that organism. In this way, assisted by enzyme promiscuity, a retrograde-type acquisition (*35*) of the Krebs cycle can be imagined, which would be aided by the environmental availability of all its constituents, and suggests an anaplerotic-like function at the outset of biology. Similarly, the door may have been opened for retrodictive learning of the ultimate and penultimate steps in other amino acid biosyntheses through a combination of the oxidative chemistry now described and by virtue of amino nitrile–cyanohydrin equilibria and hydrolysis (*2*). It is of note that the same photooxidative chemistry, which leads to Krebs cycle intermediates, is also predisposed to form malonate **17** and 3-hydroxypropionate **28** from malate **10** and propionate **19** and 4-hydroxybutyrate **20** from α-hydroxyglutarate **18**, meaning that the bones of all four early CO_2_ fixing autocatalytic cycles (reverse Krebs cycle, 3-hydroxypropionate bicycle, 3-hydroxypropionate/4-hydroxybutyrate cycle, and dicarboxylate/3-hydroxypropionate cycle) could have been laid down by a common chemistry (*36*).

## MATERIALS AND METHODS

### General experimental

Reagents and solvents were bought from Sigma-Aldrich, Alfa Aesar, and Santa Cruz Biotechnology and were used without further purification. Reagents were weighed using a Sartorius M-pact AX124 balance. Photochemical reactions were carried out in Spectrosil quartz cuvettes with four windows using a Rayonet RPR-200 photochemical reactor chamber, with cooling fans switched on and fitted with low-pressure Hg lamps purchased from Rayonet (RPR-2537A; principal emission, 254 nm). Internal temperature of the reactor was ~40°C. A Mettler Toledo SevenMulti pH/mv module fitted with a Thermo Fisher Scientific Orion 8103BN pH probe was used to measure pH, and deoxygenation of solvents was achieved by sparging with argon for 20 to 30 min before use. This included solutions of HCl and NaOH that were only used for adjusting the pH of the reaction solutions, and so, alteration of the acidity/basicity was of no concern. During pH measurement/adjustment, the samples were exposed to the atmosphere for 30 s or less. ^1^H NMR spectra were acquired using Bruker Ultrashield 400 Plus or Bruker Ascend 400 (at 400.1 MHz) using HOD/H_2_O suppression to collect ^1^H NMR data (reactions were run in 10% D_2_O in H_2_O solutions). Quantitative ^13^C NMR spectra were acquired on a Bruker Avance-II spectrometer with broadband detect cryogenic probe at a ^13^C frequency of 125.7 MHz and a sample temperature of 298 K. Carbon NMR signals were quantified from a ^13^C NMR spectrum with inverse gated decoupling, irradiating ^1^H during the acquisition period only, to eliminate {^1^H}-^13^C heteronuclear nuclear Overhauser effect. A total of 3800 scans were recorded using a 30° ^13^C pulse flip angle and 60-s pulse recycle delay to ensure complete relaxation of all spins between pulses. Spectra were acquired and processed using TopSpin version 3.2 software. Coupling constants (*J*) are given in hertz, and the notations s, d, t, app.t, and m represent the multiplicities singlet, doublet, triplet, apparent triplet, and multiplet, respectively. Chemical shifts (δ) are given in parts per million. Yields of reactions were determined by relative integration of the signals in ^1^H NMR spectra of crude reaction mixtures, inclusion of an inert standard, or spiking reaction mixtures with an external standard of known concentration.

### General procedures

#### *Procedure 1: Oxidation of* α*-hydroxy carboxylates*

Na_2_HPO_4_ (28 mg, 0.200 mmol) and the α-hydroxy acid (0.120 mmol) were dissolved in degassed 10% D_2_O in H_2_O (1 ml), and then the required amount of NaSH.*x*H_2_O (>60%) was added. The solution was adjusted to pH 6.5 with degassed NaOH/HCl, and then the volume was made up to 2 ml using degassed 10% D_2_O in H_2_O. The solution was transferred to a cuvette, capped, and irradiated for the desired amount of time before being examined by NMR spectroscopy.

#### *Procedure 2: Oxidation of* α*-hydroxy carboxylates in the presence of HCN*

Na_2_HPO_4_ (21 mg, 0.150 mmol), KCN (6 mg, 0.090 mmol), and the α-hydroxy acid (0.120 mmol) were dissolved in degassed 10% D_2_O in H_2_O (1 ml), and then the required amount of NaSH.*x*H_2_O (>60%, 5 mg, 0.050 mmol) was added. The solution was adjusted to pH 6.5 with degassed NaOH/HCl, and then the volume was made up to 2 ml using degassed 10% D_2_O in H_2_O. The solution was transferred to a cuvette, capped, and irradiated for the desired amount of time before being examined by NMR spectroscopy.

#### *Procedure 3: Oxidation of* α*-hydroxy carboxylates in the presence of excess HCN*

Na_2_HPO_4_ (28 mg, 0.200 mmol), KCN (13 mg, 0.200 mmol), and the α-hydroxy acid (0.120 mmol) were dissolved in degassed 10% D_2_O in H_2_O (1 ml), and then the required amount of NaSH.*x*H_2_O (>60%, 5 mg, 0.050 mmol) was added. The solution was adjusted to pH 6.5 with degassed NaOH/HCl, and then the volume was made up to 2 ml using degassed 10% D_2_O in H_2_O. The solution was transferred to a cuvette, capped, and irradiated for the desired amount of time before being examined by NMR spectroscopy.

#### Procedure 4: Recovery of carbonyl compounds from cyanohydrins

The carbonyl compound (0.030 mmol) and KCN (0.040 mmol) were dissolved in H_2_O (20 ml), and the pH was adjusted to 6.5 to make the cyanohydrin. Nitrogen was bubbled through the solution for 3 days, at which point the volume was ~3 ml, and an ^1^H NMR spectrum was collected. For glyoxylate, after 3 days, the volume was made up to 20 ml once again with H_2_O, and N_2_ was bubbled through the solution for a further 3 days.

## SUPPLEMENTARY MATERIALS

Supplementary material for this article is available at http://advances.sciencemag.org/cgi/content/full/7/33/eabh3981/DC1
